# The Surface Characterisation of Fused Filament Fabricated (FFF) 3D Printed PEEK/Hydroxyapatite Composites

**DOI:** 10.3390/polym13183117

**Published:** 2021-09-15

**Authors:** Krzysztof Rodzeń, Mary Josephine McIvor, Preetam K. Sharma, Jonathan G. Acheson, Alistair McIlhagger, Mozaffar Mokhtari, Aoife McFerran, Joanna Ward, Brian J. Meenan, Adrian R. Boyd

**Affiliations:** 1 School of Engineering, Ulster University, Shore Road, Newtownabbey BT37 0QB, UK; mj.mcivor@ulster.ac.uk (M.J.M.); p.sharma@lboro.ac.uk (P.K.S.); j.acheson@ulster.ac.uk (J.G.A.); a.mcilhagger@ulster.ac.uk (A.M.); m.mokhtari@ulster.ac.uk (M.M.); mcferran-a4@ulster.ac.uk (A.M.); je.ward@ulster.ac.uk (J.W.); bj.meenan@ulster.ac.uk (B.J.M.); 2 Department of Chemical Engineering, Loughborough University, Loughborough LE11 3TU, UK

**Keywords:** additive manufacturing, advanced composite materials, 3D printing, fused filament fabrication, PEEK, polyetheretherketone, hydroxyapatite, XPS, ToFSIMS, in vitro

## Abstract

Polyetheretherketone (PEEK) is a high-performance thermoplastic polymer which has found increasing application in orthopaedics and has shown a lot of promise for ‘made-to-measure’ implants via additive manufacturing approaches. However, PEEK is bioinert and needs to undergo surface modification to make it at least osteoconductive to ensure a more rapid, improved, and stable fixation that will last longer in vivo. One approach to solving this issue is to modify PEEK with bioactive agents such as hydroxyapatite (HA). The work reported in this study demonstrates the direct 3D printing of PEEK/HA composites of up to 30 weight percent (wt%) HA using a Fused Filament Fabrication (FFF) approach. The surface characteristics and in vitro properties of the composite materials were investigated. X-ray diffraction revealed the samples to be semi-crystalline in nature, with X-ray Photoelectron Spectroscopy and Time-of-Flight Secondary Ion Mass Spectrometry revealing HA materials were available in the uppermost surface of all the 3D printed samples. In vitro testing of the samples at 7 days demonstrated that the PEEK/HA composite surfaces supported the adherence and growth of viable U-2 OS osteoblast like cells. These results demonstrate that FFF can deliver bioactive HA on the surface of PEEK bio-composites in a one-step 3D printing process.

## 1. Introduction

There is increasing interest in the use of Polyetheretherketone (PEEK) in orthopaedic implant devices due to its excellent biocompatibility, its radiolucency, chemical resistance, sterilizability, ability to be easily processed, and its favourable mechanical properties (in comparison to human cortical bone) [1,2]. To date it has found applications in spinal fusions cages, dental implants, and maxillofacial reconstruction [1,3,4]. However, despite its obvious potential in load bearing orthopaedics and reconstructive surgery, a major clinical concern is that PEEK is bioinert and that it will not provide a suitable interface for driving successful osseointegration, in vivo [2,5].

Various approaches have been suggested to enhance the surface bioactivity of PEEK, including surface modification via chemical or plasma treatment [6], coating of the surface via plasma spraying [7], sputtering [8,9], or by the direct fabrication of a composite material containing bioactive agents via injection moulding [10] or direct 3D printing [11,12]. A range of different bioactive agents have been studied as additives for PEEK based composites, namely the likes of hydroxyapatite [HA—Ca_10_(PO_4_)_6_(OH)_2_], [13] strontium (Sr) substituted apatites [14], fluoro-hydroxyapatite [15], β-tricalcium phosphate [β-TCP Ca_3_(PO_4_)_2_] [16], calcium silicate [17,18], bioglass [19], and titanium dioxide [TiO_2_] [20]. Although these different additives all offer obvious advantages when added to the PEEK matrix to create a composite material, the use of these materials could potentially alter bone homeostasis and depending on their concentration and scale (micro versus nanoparticles) they could prove toxic to osteoblasts and this needs to be considered when designing the composite material of choice [13,14,15,16,17,18,19,20].

The development of bioactive PEEK-based composite materials provides several obvious advantages over other approaches, most notably that composite materials can have tunable mechanical properties for specific applications along with the added benefit of enhanced bioactivity on its surface, enhancing its osseointegration. A range of direct processing techniques have been utilized to deliver such composite materials, which includes compounding and injection moulding [18], extrusion free forming in combination with compression moulding [21], selective laser sintering [22], cold press sintering [23], hot pressing [24], and electrostatic bonding [25]. Of these different approaches, injection moulding is still one of the most used techniques [2]. The main shortcoming of injection moulding, and the other techniques highlighted here for manufacturing PEEK and PEEK based composites with bioactive agents is the lack of flexibility for making parts with complicated geometry and the significant waste that can be generated [10]. One way to get around these inherent issues is to develop an additive manufacturing approach to creating composite materials. To date there are a significant number of reports in the literature highlighting how 3D printing can be used to develop pure PEEK and PEEK-based composites implant devices using either Selective Laser Sintering (SLS) [22,26,27] or Fused Filament Fabrication (FFF) [5,11,12] However, the SLS approach comes with a high cost, requires significant safety measures and results in significant waste of the feedstock powder, which cannot be re-used for implant preparation thereafter due to the potential for contamination. In comparison, FFF utilises a continuous filament during 3D printing which produces minimal waste and is accessible due to its relatively low cost. Furthermore, FFF printers can be easily and cheaply modified and upgraded to develop printers better suited for an intended application area. FFF does however have significant shortcomings, such as poor mechanical properties in the resultant 3D printed structures in the Z direction. PEEK has a very high melting temperature (343 °C) when compared to other biomedical polymers that can be 3D printed such as Polylactic Acid (typically between 130–180 °C). This means that when either 3D printing PEEK or a PEEK-based composite the chamber, the print bed and hot-end temperatures need to be high, typically well beyond those available in normal commercial FFF printers [28,29,30,31]. As such, obtaining the necessary properties in the 3D printed PEEK or PEEK-based composites will depend upon delivering the appropriate processing parameters during printing. Previous novel work, believed to be one of the first study of its kind, presented the properties of PEEK and hydroxyapatite (HA) composites manufactured by directly 3D printing (using FFF) from a composite PEEK/HA composite (between 0–30 wt% HA) with respect to their thermal properties, mechanical properties, surface morphology and crystallinity, and highlighted how the properties of the PEEK/HA composites could be produced to be in line with human cortical bone [11]. Several other reports have also been published showing how bioactive PEEK/apatite composites can be manufactured using FFF 3D, albeit using significantly different processing parameters and 3D printers [5,12]. Despite this, the published works is still very limited in this area.

One deficiency in the previous studies was the lack of surface analyses undertaken on the 3D printed PEEK/HA composites, which is a critical factor when considering such materials for use in implant devices in vivo. In this novel paper, we would like to address this issue and highlight further the significant developments in the direct 3D printing of PEEK/HA composites using an FFF approach. The key aim of the work was to prove that FFF 3D printing could deliver PEEK/HA composites with controllable concentrations of HA on the surface of the 3D printed structures without the need for any further processing steps to expose the bioactive HA materials (in essence a one-step process to producing bioactive PEEK/HA composites). To the best of our knowledge, this is the first time this has been reported in the literature using advanced surface characterisation techniques as detailed below. In this work we report on the direct 3D printing of the extruded PEEK/HA composite filaments via an FFF approach using a custom modified commercial printer Ultimaker 2+ (UM2+). The 3D printer was modified to operate at higher temperatures (as detailed in the Materials and Methods Section), allowing the properties of the printed PEEK bodies to be customised accordingly, namely the uppermost surface in this case. The 3D printed specimens were then subject to extensive surface characterisation regime via physical, and chemical techniques (as detailed in the Materials and Methods Section), along with an in vitro study (using U-2 OS osteoblast-like bone cells), to ascertain the potential for FFF to be a go-to manufacturing technique to produce PEEK/HA composites for orthopaedic implant devices where direct bone apposition between the implant surface and human bone is crucial for their long-term success.

## 2. Materials and Methods

### 2.1. Materials and Processing Conditions

**Figure 1:**
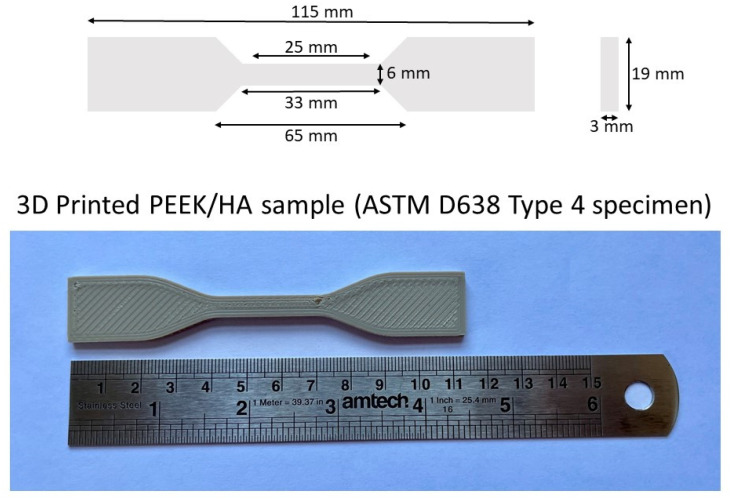
Dimensions of 3D printing sample of PEEK/HA composites in accordance with ASTM D638 Type 4 specimens.

The CAPITAL^®^R (Plasma Biotal, Buxton, England, UK) unsintered Hydroxyapatite powder (HA) was mixed with medium viscous Polyetheretherketone (PEEK) VESTAKEEP^®^ 2000P (Evonik, Essen, Germany) and processed by twin-screw extrusion to obtain continuous filaments with five different HA contents of: 0, 5, 10, 20, and 30 weight percent (wt%) HA in PEEK and a continuous diameter of 1.75 ± 0.10 mm, enabling the filament to be rolled onto a reel for 3D printing. PEEK and HA powders were dried in an oven at 170 °C for 12 h before use. A co-rotating twin-screw extruder Thermo Fischer Rheomex PTW16/40 OS (Karlsruhe, Germany) was utilised to compound the PEEK and HA into filaments as per previous studies [11]. These materials were used for 3D printing on a modified UM2+ (Ultimaker, Utrecht, The Netherlands) supplied with an all-metal hot end capable of reaching temperatures of up to 420 °C, with the heating bed allowing regulation of the temperature up to 350 °C, and heating lamps for regulation of the chamber temperature up to 230 °C, as has been described in previous work [11]. All specimens were printed as solid, fully filled structures in dog-bone shapes in accordance with the American Society for Testing and Materials (ASTM) standard D638 Type 4, as highlighted in Figure 1. Printing conditions were utilised (as optimised in previous studies) [11,12] for each sample to avoid warping of the sample, with the printing parameters described in Table 1.

### 2.2. Physical and Chemical Characterisation of the 3D Printed Samples

The 3D printed samples were analysed using X-ray Diffraction (XRD), Fourier Transform Infrared Spectroscopy (FTIR), Scanning Electron Microscopy (SEM), X-ray Photoelectron Spectroscopy (XPS), Time-of-Flight Secondary Ion Mass Spectrometry (ToFSIMS), and were also subjected to a range of in vitro characterisation techniques. All details of these analyses are provided below.

#### 2.2.1. Fourier Transform Infrared Spectroscopy (FTIR)

Fourier Transform Infrared spectroscopy (FTIR) measurements were performed using a Varian 610-IR microscope system (Santa Clara, CA, USA) with a germanium Attenuated Total Reflectance (ATR) accessory. Samples were studied in absorbance mode from 2000–800 cm^−1^, at a resolution of 4 cm^−1^, with 50 scans per sample.

#### 2.2.2. X-ray Diffraction (XRD)

X-ray diffraction (XRD) measurements were performed using an Empyrean diffractometer (Malvern Panalytical, Malvern, UK) operating at 45 kV and 40 mA using a Cu K_α_ radiation (λ = 1.54187 Å). Diffractograms were measured over the range of 2-Theta (θ) from 5° to 60° with an angular step interval of 0.0394°.

#### 2.2.3. Scanning Electron Microscopy (SEM)

Scanning electron microscopy (SEM) analysis of the samples was performed using a Hitachi SU5000 field emission instrument (Krefeld, Germany). The SEM images were measured in Backscattered Electron mode (BSE) at low vacuum, whereby the samples were not coated with a conductive layer. The accelerating voltage used was 10KV and images were collected at a pressure of 50 Pa. The low magnification SEM images illustrating the print line diameters were measured using the same SEM system in Backscattered Electron mode (BSE), whereby the samples were not coated with a conductive layer. Analyses was performed on the side of the dog-bone samples.

#### 2.2.4. Stylus Profilometry

The surface roughness (R_a_) of the different 3D printed samples were determined using a Dektak 8 stylus profilometer (Veeco Instruments Inc, Plainview, NY, USA). A 2.0 µm diameter diamond tipped stylus was employed with scans lengths of 2500 µm at a resolution of 0.278 µm/pt, at with a load of 5 mg. The surface roughness was measured on the as-received top surface taken along the parallel length of the 3D printed dog-bone samples. Statistical measurements were performed using MS Excel 2016 for Windows (Microsoft, Redmond, WA, USA). The roughness values of the different 3D printed samples (0, 5, 10, 20, and 30 wt% HA) are reported as the mean ± standard deviation value (where n = 4. A one-way analysis of variance (ANOVA) was applied to test for statistically significant differences between the sample types with a value of *p* < 0.05 considered to be statistically significant. The Bonferroni multiple test comparison test was applied to compare values between successive pairs of sample types.

#### 2.2.5. X-ray Photoelectron Spectroscopy (XPS)

X-ray Photoelectron Spectroscopy (XPS) analyses were performed using an Axis Ultra DLD Spectrometer (Kratos, Manchester, UK). Spectra were analysed using monochromated Al K_α_ X-rays (hѵ = 1486.6 eV) operating at 5 mA and 15 kV. All high-resolution spectra for C1s, O1s, Ca2p, and P2p were recorded at a pass energy of 20 eV. Sample charging was corrected by setting the lowest BE component of the C1s spectral envelope to 285.0 eV [8]. Photoelectron spectra were further processed by subtracting a Tougaard background and using the peak area for the most intense spectral line of each detected elemental species to determine the percent of atomic concentration. In total, 3 areas were analysed from each sample. Peak-fitting was carried out using a mixed Gaussian–Lorentzian synthetic peak function using Casa software (version 2.3.19PR1.0) (Casa Software Ltd., England, UK).

#### 2.2.6. Time-of-Flight Secondary Ion Mass Spectrometry (ToFSIMS)

Time-of-Flight Secondary Ion Mass Spectrometry (ToFSIMS) data was obtained using a ToF-SIMS V instrument (IONTOF, Münster, Germany) equipped with a 25 keV bismuth (Bi) liquid metal ion gun (primary ion source) with an emission current of 1 microampere (μA) and a pulsed target current of 14 nanoamperes (nA). A pressure of at least 5.00 × 10^−8^ Pa was maintained in the analysis chamber throughout experimentation. Data was collected by using the Bi^1+^ primary ion gun species operating in both the positive and negative polarity. ToF-SIMS ion intensity images of 256 × 256 pixels were acquired using a random raster, using spectroscopy mode over a 500 μm^2^ area on the sample surface. An electron flood gun was used to shower the sample with electrons to prevent a build-up of charge was operated at a filament current of 2.35 A during acquisition. Data acquisition, processing and analysis was performed using Surface Lab 6 (IONTOF, Münster, Germany).

#### 2.2.7. Contact Angle

Static contact angle (CAM 2000, KSV Instruments Ltd., Espoo, Finland) was used to determine changes in the surface wettability of the top surface of the samples after 3D printing. The sessile drop method with water as the liquid phase was used, operating with a 5 μL droplet on each substrate surface. Statistical measurements were performed using MS Excel 2016 for Windows (Microsoft, Redmond, WA, USA). The static contact angle of the different 3D printed samples (0, 5, 10, 20, and 30 wt% HA) are reported as the mean ± standard deviation value (where n = 6. A one-way analysis of variance (ANOVA) was applied to test for statistically significant differences between the sample types with a value of *p* < 0.05 considered to be statistically significant. The Bonferroni multiple test comparison test was applied to compare values between successive pairs of sample types.

### 2.3. In Vitro Characterisation of the 3D Printed Samples

The samples were also subjected to a range of in vitro characterisation techniques. Protocols and experimental techniques are discussed below.

#### 2.3.1. Scaffold Sterilisation

Individual scaffold types were securely wrapped in triple-layered aluminium foil before being dry sterilised overnight in a dry oven (OV-12, Thermo Fisher Scientific, Waltham, MA, USA) at 135 °C; a temperature chosen to prevent crossing the glass transition temperatures of PEEK. Under aseptic conditions, sterile scaffolds were transferred to sterile 12-well tissue culture plastic (TCP) plates until required.

#### 2.3.2. Cell Culture and Cell-Seeding

Human osteosarcoma cells from an immortalised cell-line, U-2 OS (HTB-96, ATCC, Manassas, VA, USA), were cultured and maintained as per the manufacturer’s instructions. Where possible cells were monitored using an inverted microscope (Nikon Eclipse TS100, Nikon, Amsterdam, The Netherlands). At day 0, cell suspensions (cells of passage no. 13) were standardised to 2.3 × 10^6^ cells per mL and 75 µL (172,500 cells per scaffold) centrally pipetted onto each scaffold followed by a 2 h incubation under standard conditions, addition of 1925 µL of medium and further incubation under standard conditions until day 7, with medium replenished every three days where possible. TCP controls were included; negative controls with medium (no cells and no scaffold) and positive controls with cells and medium (no scaffold) and treated in the same manner as that of the test scaffolds described above.

#### 2.3.3. Measuring Cell Metabolic Activity

At day 7 metabolic activity of any cell attachment on each scaffold type was obtained using a 3-(4,5-dimethylthiazol-2-yl)-2,5-diphenyltetrazolium bromide) MTT colorimetric assay. Test scaffolds, controls and their medium were moved to a new 12-well TCP plate. MTT solution, 5 mg/mL in 0.01 M PBS, was added to all wells (at 10% of the total well volume) followed by incubation for 2 h at 37 °C in the dark. Any formazan crystals were observed and then dissolved in 10% SDS with 0.01 M HCl followed by incubation for 1.5 h at 15 RPM at room temperature. Following thorough mixing of the wells, aliquots (3 × 100 µL per well) were transferred to a clear 96-well microplate and their absorbance at a wavelength of 562 nm measured using a Tecan Spark Spectrophotometer (Tecan Group Ltd., Männedorf, Switzerland). Each scaffold type was tested in triplicate. All reagents were from Sigma-Aldrich (Merck, Kenilworth, NJ, USA).

#### 2.3.4. Measuring Deoxyribonucleic Acid (DNA)

At day 7, a cumulative DNA concentration for any cell attachment on each scaffold type was obtained using a commercial kit, Quant-iT™ Pico Green™ dsDNA (PG) Assay Kit (P7581, Thermo Fisher Scientific, Waltham, MA, USA). Medium was aspirated from wells of test scaffolds and controls followed by their move to a new 12-well TCP plate from where wells were washed twice with 0.01 M PBS and any attached cells detached by incubating in TrypLE express (Thermo Fisher Scientific) with 1% Triton X-100 (Sigma-Aldrich, St. Louis, MO, USA) under standard conditions for 30 min. The cell suspension was transferred to a sterile microtube which was then frozen and thawed twice.

Using supplied kit reagents and following the manufacturer’s instructions, five DNA standards, 1000, 100, 10, 1, and 0 ng/mL, for use as a standard curve, were prepared along with PG fluorescent probe, using supplied TE buffer (at 1×) as the diluent. PG fluorescent probe was added to DNA within both the standards and samples in a 1:1 volume ratio mix, thoroughly mixed and incubated for 5 min at room temperature in the dark prior to transfer to a black 96-well microplate (3 × 200 µL per standard and sample). Fluorescent measurements (in triplicate per standard and sample) were taken at an excitation wavelength of 480 nm and an emission wavelength of 540 nm with a Tecan Genios Spectrophotometer (Tecan Group Ltd., Switzerland). Fluorescence (arbitrary units) for standards was plotted against their known DNA concentrations (ng/mL) with the resulting standard curve equation used to calculate unknown DNA concentrations, an average DNA concentration (in ng/mL) obtained per scaffold type (Microsoft Excel^®^, Microsoft Inc., Redmond, WA, USA).

#### 2.3.5. Scanning Electron Microscope (SEM) of the Osteoblast-Like Cells

At day 7, medium was aspirated from wells of test scaffolds and controls followed by their move to a new 12-well TCP plate. Wells were washed twice with 0.01 M PBS and thrice with deionised water followed by chemical fixing using Karnovsky’s reagent (Polysciences, Inc., Warrington, PA, USA) (5% glutaraldehyde/3.2% PFA for 8 min at room temperature and then washed twice with 0.01 M PBS. Gradual dehydration was performed using an alcohol series of increasing ethanol concentration, 25, 50, 75, 90, and 100% ethanol, for 8 min each at room temperature, followed by volume ratio mix of 100% ethanol/100% hexamethyldisilizane (HMDS) for 8 min at room temperature. Wells were chemically dried overnight at room temperature using 2–3 drops of 100% HMDS. All reagents were from Sigma-Aldrich.

To maximise image quality, scaffolds were coated with a thin layer of gold-palladium (18 nm) using Emitech K500X sputtering system (Quorum Technologies, Lewes, UK) at 25 mA for 150 s. Field emission scanning electron microscopy, FESEM (SU5000, Hitachi, Tokyo, Japan), was used to study surface topography at 5 KV voltage from three random locations per cell-seeded scaffold type.

#### 2.3.6. Statistical Analyses for the In Vitro Measurements

Statistical analyses for the in vitro measurements, where n = 3 (minimum), were performed using GraphPad Prism version 8 for Windows (GraphPad Software, San Diego, CA, USA). Any statistically significant difference in results for cells seeded and cultured on pure PEEK scaffold to those on the PEEK/HA variant scaffolds (with 5, 10, 20 and 30 wt% HA) was determined using Dunnett’s multiple comparison test with a value of *p* < 0.05 taken as statistically significant.

## 3. Results

### 3.1. Physical and Chemical Characterisation of the 3D Printed Samples

#### 3.1.1. FTIR

**Figure 2:**
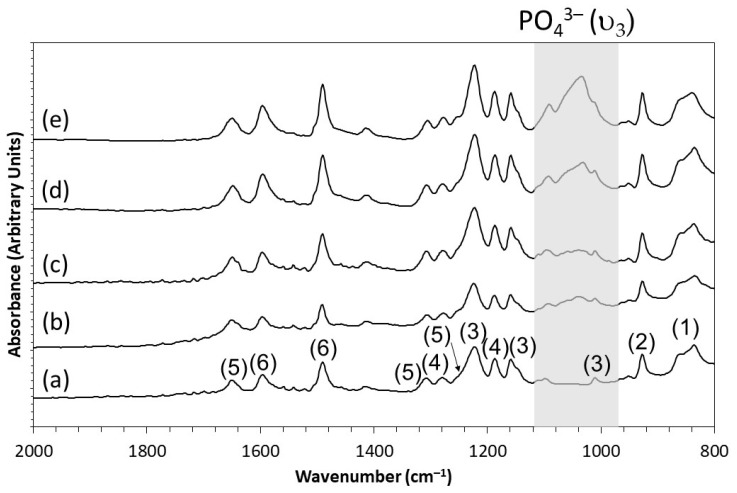
FTIR spectra for (**a**) 0HA, (**b**) 5HA, (**c**) 10HA, (**d**) 20HA, and (**e**) 30HA. Key for figure: (1)—Out of plane, aromatic hydrogen, (2)—Diphenyl ketone band, (3)—In plane, aromatic hydrogen, (4)—Diphenyl ether groups, (5)—Carbonyl group, and (6)—Skeletal phenyl ring. The key PO_4_^3−^ (υ_3_) peaks of HA are highlighted in the grey shaded area.

The results from the FTIR analyses are shown in Figure 2. For the 3D printed pure PEEK sample (0HA), several peaks were observed in the FTIR spectrum (Figure 2a), including the carbonyl stretching vibrational modes at 1655, 1485, 1415 cm^−1^, and a corresponding shoulder at 1252 cm^−1^. The broad band observed at 1200 cm^−1^ corresponds to the vibration of aromatic ether (C–O–C). Skeletal in plane phenyl ring vibrations are also clearly shown in Figure 2a at 1594, 1485, and 1414 cm^−1^. The bending motion of the carbonyl group (C–C(=O)–C) can be observed at 1307 cm^−1^, with the asymmetric stretching of the diphenyl ether group highlighted by the peaks at 1277 and 1189 cm^−1^. The aromatic hydrogen bending deformations are also clearly shown at 1218, 1157, and 1009 cm^−1^. A diphenyl ketone band is observable at 925 cm^−1^ and the out of plane bending modes of the aromatic hydrogens are located at 852 and 837 cm^−1^. These may be attributed to the phenyl ring (C–H) deformation. All the absorbance bands observed in Figure 2a for the 0HA sample are in line with those expected for PEEK [18]. No peaks corresponding to hydroxyapatite, namely the PO_4_^3−^ (υ_3_) (typically around 1200–900 cm^−1^), are observed for the 0HA sample as highlighted in Figure 2a [32]. In comparison, the peak positions and peak intensities for PEEK remain unchanged for the 3D printed PEEK/hydroxyapatite composites and can be observed in Figure 2b–d for the 5HA, 10HA, 20HA, and 30HA samples, respectively. However, the presence of the PO_4_^3−^ (υ_3_) vibrational bands can be clearly seen, with increasing peak intensity as the concentration of hydroxyapatite increases in the samples.

#### 3.1.2. XRD

**Figure 3:**
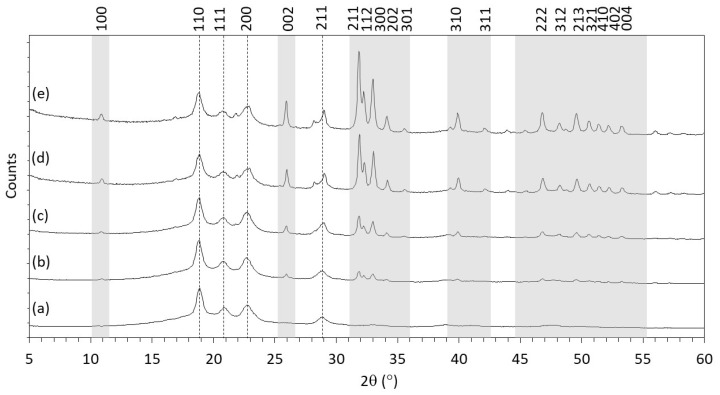
XRD patterns for (**a**) 0HA, (**b**) 5HA, (**c**) 10HA, (**d**) 20HA, and (**e**) 30HA. The main peaks for PEEK are given by dashed lines, the main peaks for HA are illustrated in the shaded areas. Miller indices for each peak are highlighted at the top of the figure.

The X-ray Diffraction (XRD) patterns for each of the PEEK/HA composites are illustrated in Figure 3. For the 0HA sample, strong peaks corresponding to various diffraction planes for PEEK have been identified at 18.86°, 20.90°, 22.76°, and 28.75° 2θ, and are attributed to orthorhombic PEEK crystal planes (110), (111), (200) and (211), respectively. [11,18] In the composites containing HA (loaded between 5–30% by weight), additional peaks were observed at 25.93°, 31.85°, 32.24, 32.98°, and 34.12° 2θ and correspond to (002), (211), (112), (300), and (202) planes of crystalline HA in accordance with International Centre for Diffraction Data (ICDD) File# 09-0432. Upon increasing the HA concentration in the PEEK/HA composites the intensities of the HA peaks increased (with respect to the PEEK), as would be expected as shown in Figure 3. No significant shifts in the peak positions were observed for any of the PEEK/HA composites.

#### 3.1.3. SEM

**Figure 4:**
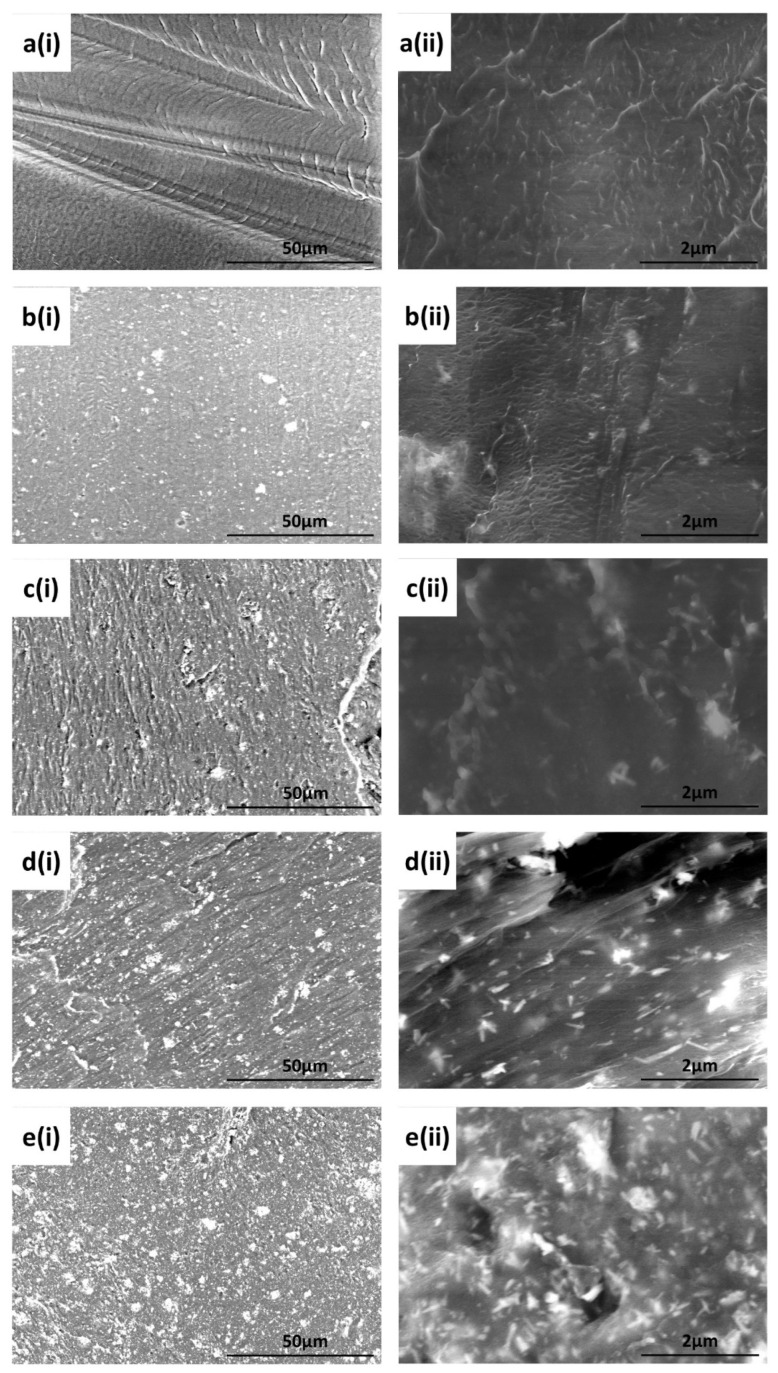
SEM images for (**a**) 0HA, (**b**) 5HA, (**c**) 10HA, (**d**) 20HA, and (**e**) 30HA. Here, set (**i**) and (**ii**) are recorded at 1 k and 20 k magnification.

Scanning Electron Microscopy (SEM) images of the 3D printed 0HA sample are shown in Figure 4a(i,ii) (at different magnifications). From these images of the native 3D printed surface, it is clear there are no particles of HA present in the pure PEEK sample, as would be expected. The sample does not have any significant surface features, lacks any porosity, and shows the presence of some fabrication lines as can be observed in Figure 4a(i). No particles, which would be indicative of HA, can be seen in Figure 4 for the pure PEEK, as would be expected. In comparison, the SEM images of the 3D printed 5HA sample (Figure 4b(i,ii)) shows the presence of HA particles, which are distributed homogeneously across the surface of the sample. These HA particles measure up to and around 5 µm in diameter, with the presence of sub-micron particles clearly present as well. No porosity can be observed in this 5HA sample. As the concentration of the HA is increased in the PEEK/HA composites up to 30 wt% HA, there is an obvious increase in the HA particles on the surface of each of the samples as can be observed in Figure 4c(i,ii)–e(i,ii) for the 10HA, 20HA, and 30HA samples, respectively. From these SEM results the HA particles are distributed homogeneously across the surface of this 3D printed samples. Again, no obvious porosity can be seen on the SEM images surface of any of these 3D printed HA/PEEK composites.

Low magnification cross-sectional SEM images of the 3D printed samples are shown in Figure S1. All 3D printed samples seem to be relatively flat, with minimal porosity observed on the printed lines, although some minor porosity is observed as the content of the HA increases in the samples (between 5 (0HA)–30% (30HA)) and may indicate a lack of adhesion as the HA content increases. The 3D printed body is built with lines, which form layers. In general, the lines from printing appear to be relatively consistent in diameter as shown in Figure S1, with values of ~100 µm for each different print composition. The lines appear to be less homogeneous with respect to their diameter for the pure PEEK (0HA) and the 5HA samples when compared to the 10–30 wt% PEEK/HA samples, as highlighted in Figure S1.

#### 3.1.4. Stylus Profilometry

The surface roughness (R_a_) of the surface of the 3D printed samples (taken along the long parallel top surface of the 3D printed samples) were determined using stylus profilometry and the results presented in Figure S2 and Table S1. Although the surface roughness values were observed to vary (between 2.70–4.40 µm), no statistically significant differences were observed from the Bonferroni Post-Hoc tests.

#### 3.1.5. XPS

**Figure 5:**
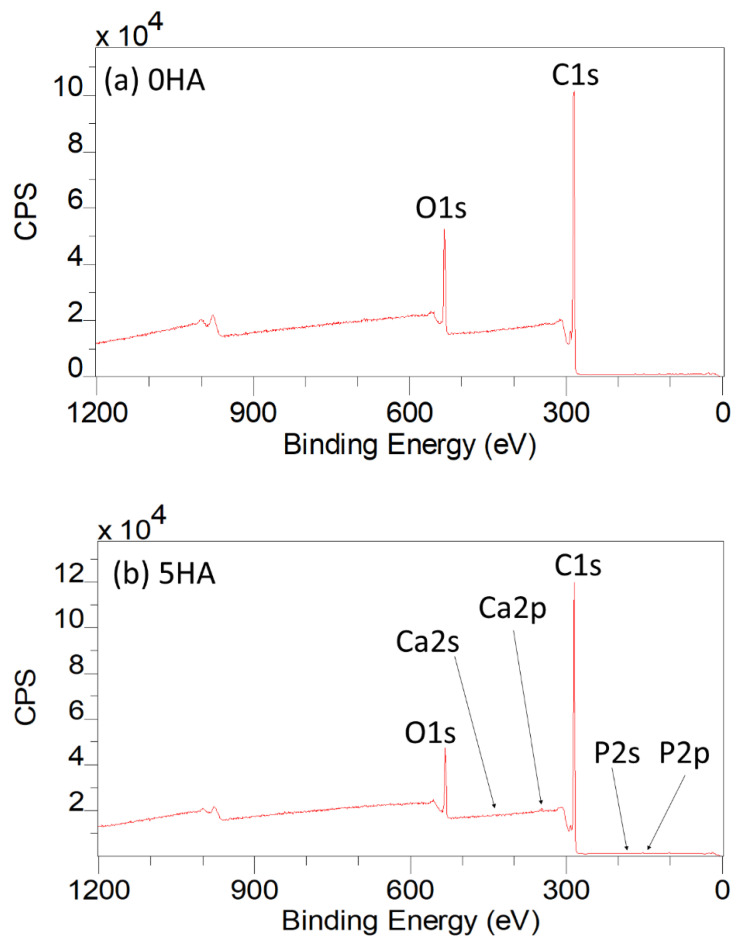
XPS wide Energy Survey Scans for (**a**) 0HA and (**b**) 5HA.

**Figure 6:**
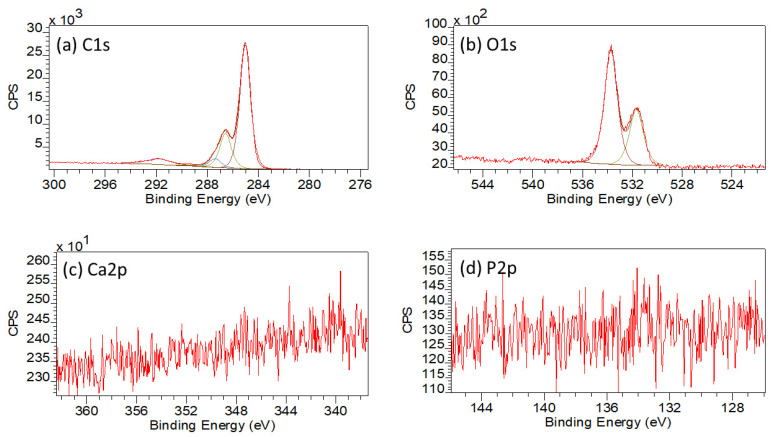
XPS high resolution scans for 0HA where (**a**) C1s, (**b**) O1s, (**c**) Ca2p, and (**d**) P2p.

The XPS results for the 3D printed 0HA sample are given in Figure 5a (Wide Energy Survey Scan) and Figure 6a–d (high resolution scans for carbon (C1s), Oxygen (O1s), calcium (Ca2p), and phosphorus (P2p), respectively). Table 2 and Table 3 highlight the peak positions and quantification results for the C1s, O1s, Ca2p, and P2p peaks, respectively. Analysis of the 0HA sample (pure PEEK) reveals that the uppermost surface (<10 nm) consisted of C and O only, no Ca or P, indicative of HA was detected. Table 3 shows the C and O concentrations (Atomic Concentration %) measured at 87.85 ± 0.76 and 12.15 ± 0.76, respectively, and an O/C ratio of 0.14 for the PEEK substrate. This compares favourably with an O/C ratio of 0.16 for PEEK as highlighted in the literature. [8,33,34,35] The high resolution C1s spectrum, as shown in Figure 6a has been peak fitted using four peaks, of which the highest intensity peak has been noted at 285.0 eV representing the C–H and C–C bonds [8,34,35,36,37]. A further peak was noted at 286.6 eV, which is known to correspond to C–O ether bonding. A peak at the higher B.E of 287.4 eV is indicative of O=C carbonyl bonds, with the weak peak at 289.2 eV indicative of O–C=O bonds or a CO_3_^2−^ species [8,34,35]. A low intensity peak at a 291.8 eV, 5 % (C4), has been attributed to a shakeup satellite, occurring due to the presence of π–π* transitions [8,34,35,37]. For the high-resolution O1s envelope (Figure 6b) revealed two peaks, the most intense peak attributable to the ether, O–C group, at 533.8 eV, whilst the peak located at 531.6 eV was found to be indicative of the carbonyl group, C=O [35,38].

**Figure 7:**
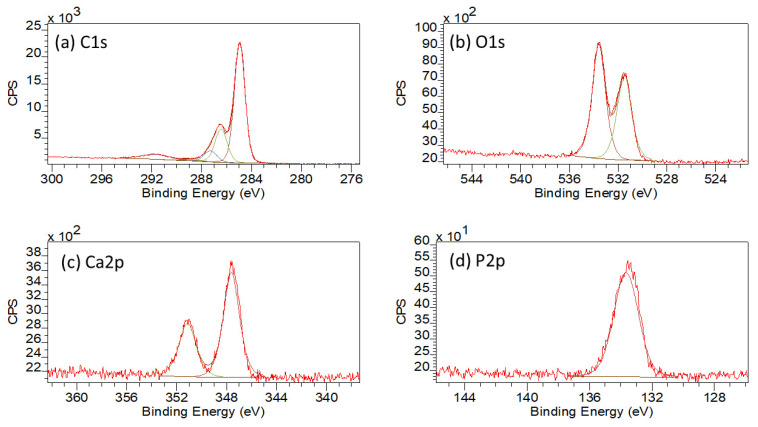
XPS high resolution scans for 30HA where (**a**) C1s, (**b**) O1s, (**c**) Ca2p, and (**d**) P2p.

In comparison, the WESS for the 5HA 3D printed sample, illustrated in Figure 5b shows the presence of weak calcium (Ca2p and Ca2s) and phosphorus (P2p and P2s) species of the surface along with carbon (C1s) and oxygen (O1s). For the 5HA sample, the high resolution C1s envelope, as shown in Supplementary Figure S3a, had three main peaks, representative of C–C/C–H, C–O, and C=O bonds at 285.0, 286.7, and 287.3 eV along with the π–π* shakeup peak at 291.8 eV (also refer to Table 2) [8,34,35,37]. No peak was observed around 289.0 eV (indicative of the O–C=O bond) as was seen for the 0HA sample. The O1s envelope (Figure S3b) displayed two distinct peaks, the most intense peak attributable to P-O bonding, with a contribution from O–C species at 533.7 eV, whilst the peak located at 531.7 eV was found to be indicative of P=O, (with a small contribution from C=O groups), as the C=O bonding was seen to diminish significantly in the corresponding C1s envelope [8]. When peak fitted, the Ca2p envelope exhibited two distinct peaks at 347.6 eV (Ca2p3/2) and 350.9 eV (Ca2p1/2) as can be seen from the high-resolution scan in Figure S3c and Table 2. The P2p peak, highlighted in Figure S3d and Table 2, shows the presence of a single peak at 133.6 eV, which will contain contributions from both the P–O and P=O species [8]. The Ca/P ratio for the surface of the 5HA sample was much higher than expected for pure HA (2.78 ± 0.87), although the O/C dropped to 0.11 when compared to the 0HA sample [32]. The peak positions reported for the 10HA, 20HA, and 30HA samples follow a similar pattern to that of the 5HA sample as highlighted in Table 2. The high-resolution scans for the 30HA sample are given in Figure 7. However, the intensity and resolution of both the Ca2p and P2p high-resolution peaks can be seen to increase significantly as the HA content increases in the composite samples, with the Ca/P ratio falling to 2.22 ± 0.81 (10HA), 1.91 ± 0.69 (20HA) and 1.59 ± 0.09 (30HA). The O/C is seen to increase slightly with increasing HA content as shown in Table 3.

#### 3.1.6. ToFSIMS

**Figure 8:**
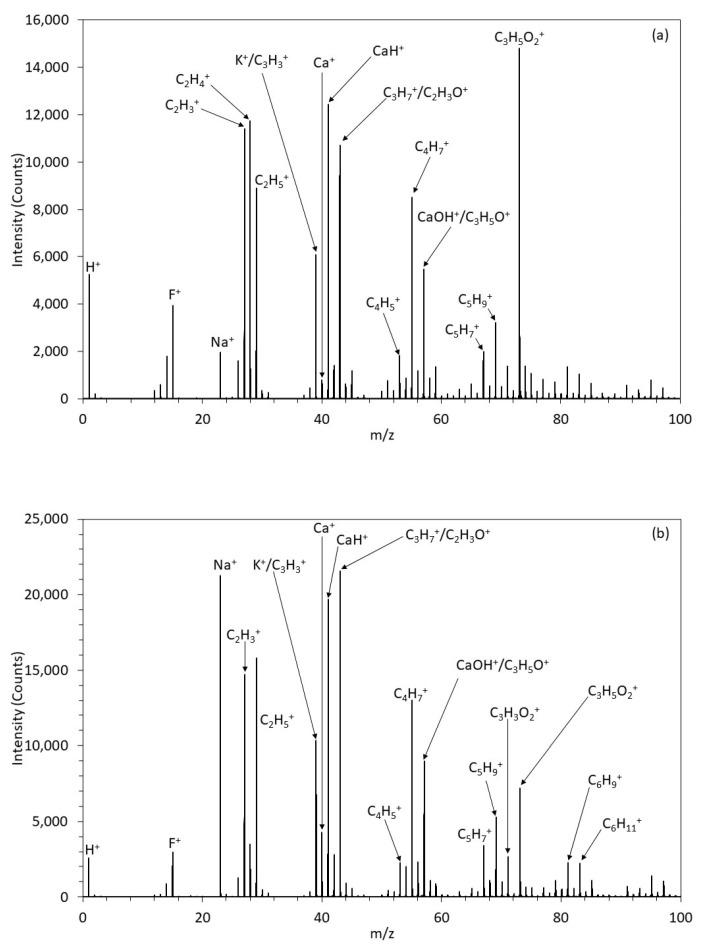
(**a**) ToFSIMS spectrum for 0HA. (**b**) ToFSIMS spectrum for 30HA.

**Figure 9:**
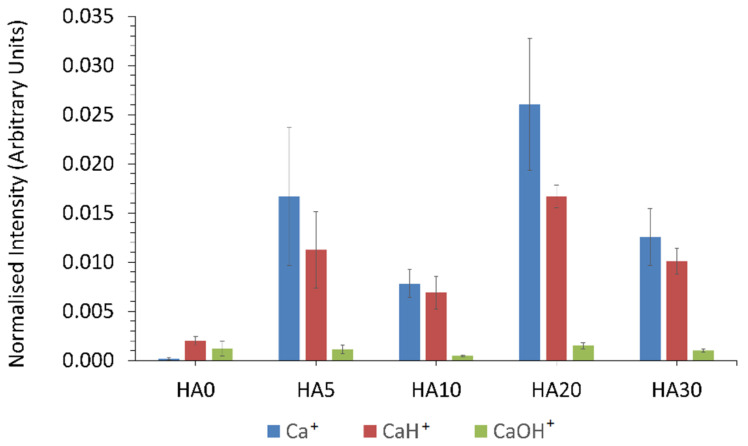
ToFSIMS normalised ion intensity for the surface of the samples.

**Figure 10:**
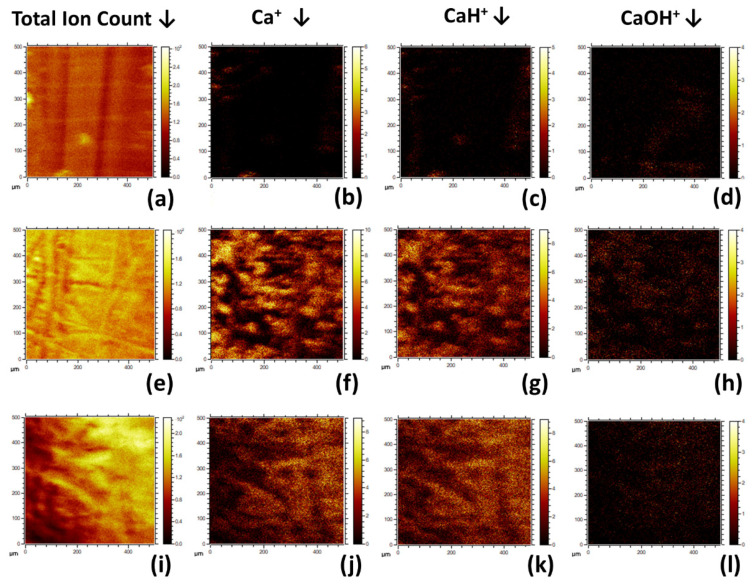
Positive Polarity ToFSIMS maps for 0HA ((**a**) Total Ion Count) ((**b**) Ca^+^), ((**c**) CaH^+^), ((**d**) CaOH^+^), 5HA ((**e**) Total Ion Count) ((**f**) Ca^+^), ((**g**) CaH^+^), ((**h**) CaOH^+^), and 30HA ((**i**) Total Ion Count) ((**j**) Ca^+^), ((**k**) CaH^+^), ((**l**) CaOH^+^). For scale, each positive ion image is 500 µm × 500 µm. The ion intensity of each image is highlighted by the scale bar located to the right-hand side of each image.

From the positive survey spectrum for the 0HA sample, as shown in Figure 8a a range of different peaks with a m/z of 15 (F^+^), 23 (Na^+^), 27 (C_2_H_3_^+^), 28 (C_2_H_4_^+^), 29 (C_2_H_5_^+^), 39 (K^+^/C_3_H_3_^+^), 40 (Ca^+^), 41 (CaH^+^), 43 (C_3_H_7_^+^/H_3_O^+^), 51 (C_4_H_5_^+^), 53 (C_4_H_7_^+^), 57 CaOH/C_2_H_5_O^+^), (67(C_5_H_7_^+^), 69 (C_5_H_9_^+^), and 73 (C_2_H_5_O_2_^+^) [8,37]. The positive ion survey spectra of the 5HA 3D printed composite sample is shown in Supplementary Figure S4 with peaks observed like that seen for the 0HA, but with many of the peaks that are indicative of organic species appearing to have lower relative intensities. A similar pattern is observed for the survey spectra for the other samples, with the HA30 shown in Figure 8b. Normalised intensities of Ca^+^, CaH^+^, and CaOH^+^ about the total ion count are presented in Figure 9. The HA0 sample surface exhibits low level peaks related to the HA apart from a small amount of calcium contamination. The 5HA surface exhibited a significant increase in Ca^+^ and CaH^+^ ion intensity, as shown in Figure 9**,** with virtually no change in intensity for CaOH^+^. All PEEK/HA samples had higher ion intensities for both Ca^+^ and CaH^+^ species, indicating that the HA materials are readily available at the uppermost surface regions of the samples, when compared to the 0HA (Pure PEEK) sample. The positive polarity ion maps, as shown in Figure 10, for the 0HA, 5HA, and 30HA 3D printed sample show low levels of calcium contamination and almost no counts for Ca^+^, CaH^+^, and CaOH^+^ ions (as shown in Figure 10b–d), respectively. Positive ion polarity maps for the 5HA (Figure 10e–h) and 30HA (Figure 10i–l), 3D printed samples show a homogenous distribution of Ca^+^ and CaH^+^ ions across the surface, with low counts reported for the CaOH^+^ species.

#### 3.1.7. Water Contact Angle

**Figure 11:**
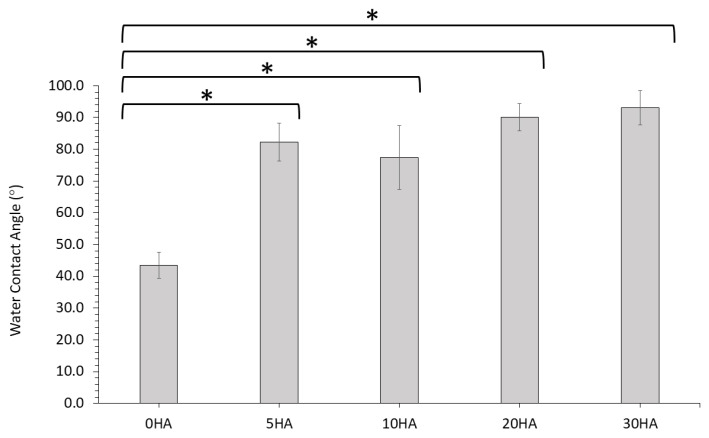
Water Contact Angle of the 3D printed samples (°). N = 6 for each sample type. Error bars represent standard deviation. (*—*p* < 0.05).

The water contact angle of all the surfaces (top surface of the 3D printed samples) of the different 3D printed samples were determined (in degrees °) and the results presented in Figure 11 and Table S2. The 0HA sample (pure PEEK) had a contact angle measurement of 43.56 ± 4.11. The values for all the 3D printed PEEK/HA composites were found to be much higher, as shown in Table S2 and Figure 11. Values were reported between 77.40 ± 10.03° − 93.08 ± 5.44° and were all found to be statistically different to the 0HA sample.

### 3.2. In Vitro Characterisation of the 3D Printed Samples

#### 3.2.1. MTT Testing for Cell Metabolism

**Figure 12:**
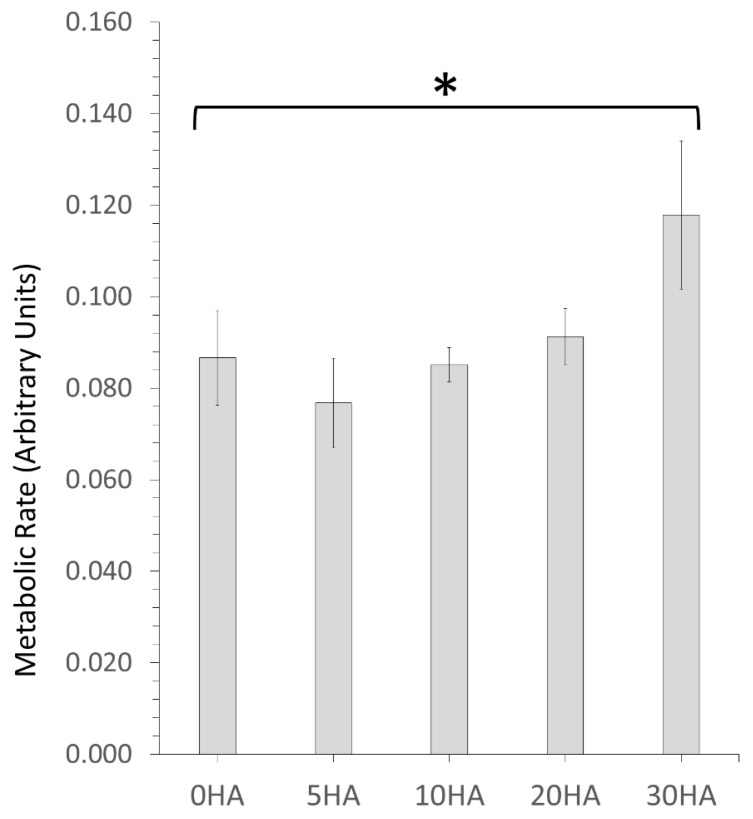
MTT assay results representing cell metabolic activity for U-2 OS cells adhered to the 3D printed samples and cultured under standard conditions for 7 days. Each sample type was tested in triplicate. Error bars represent standard deviation. (*—*p* < 0.05).

Cellular metabolic activity was determined at day 7 using MTT assay which measured the ability of mitochondrial de-hydrogenase enzymes to convert the soluble yellow MTT salt to insoluble purple formazan salt, within each set of triplicate substrate samples. The results are shown in Figure 12 and clearly show that every 3D printed sample from 5HA–20HA had similar levels of cellular metabolic activity in comparison to the 0HA control (pure PEEK 3D printed sample) with no statistically significant differences observed. However, the level of cellular metabolism was clearly higher for the 30HA sample when compared to the 0HA sample with the difference being statistically significant (*p* < 0.05).

#### 3.2.2. Pico Green™ Assay for Measuring DNA Concentration

**Figure 13:**
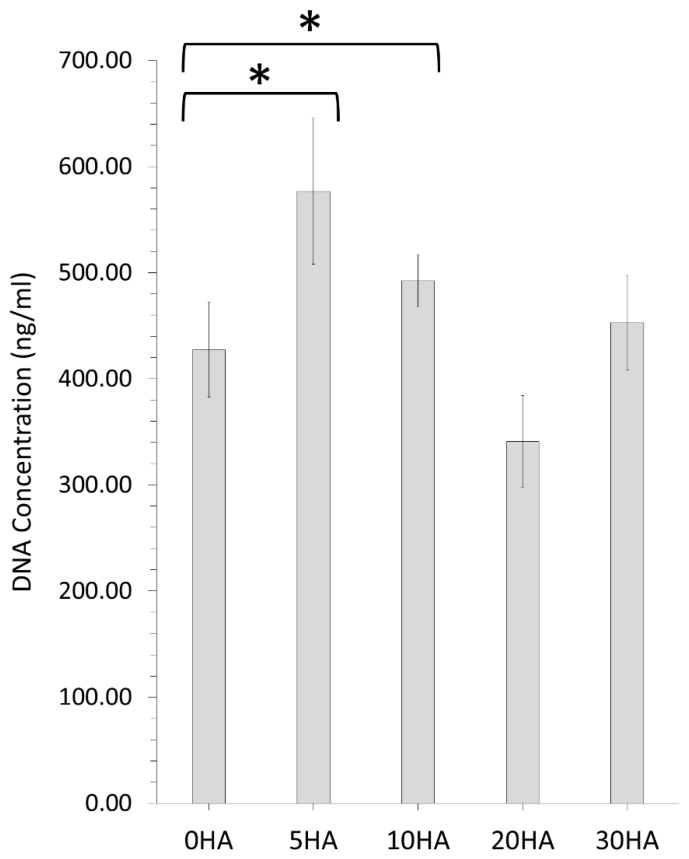
PicoGreen™ assay results representing DNA concentration for U-2 OS cells adhered to the 3D printed samples and cultured under standard conditions for 7 days. Each scaffold type was tested in triplicate. Error bars represent standard deviation. (*—*p* < 0.05).

The Quant-iT™ Pico Green™ (PG) dsDNA Assay Kit (ThermoFisher, Waltham, MA, USA), was used to detect and quantify the concentration of DNA within each set of triplicate substrate samples to assess cell viability. Figure 13 summarises the PG DNA concentration (ng/mL) values for all the different 3D printed samples after 7 days. The 0HA control 3D printed samples showed good cell viability with a DNA concentration of 427 ± 45 µg/mL. In comparison, the 5HA sample had a statistically higher DNA concentration (577 ± 69 µg/mL) than the 0HA (*p* < 0.05), indicating enhanced cell viability and proliferation at day 7 day. A similar result was observed for the 10HA, with a statistically higher DNA concentration (492 ± 24 µg/mL) than the 0HA sample as illustrated in Figure 13 (*p* < 0.05). Both the 20HA and 30HA 3D printed samples did not show any statistically different results in comparison to the 0HA sample, with DNA concentrations of 341 ± 43 µg/mL and 453 ± 44 µg/mL, respectively.

#### 3.2.3. SEM Analysis of the Cell Morphology

**Figure 14:**
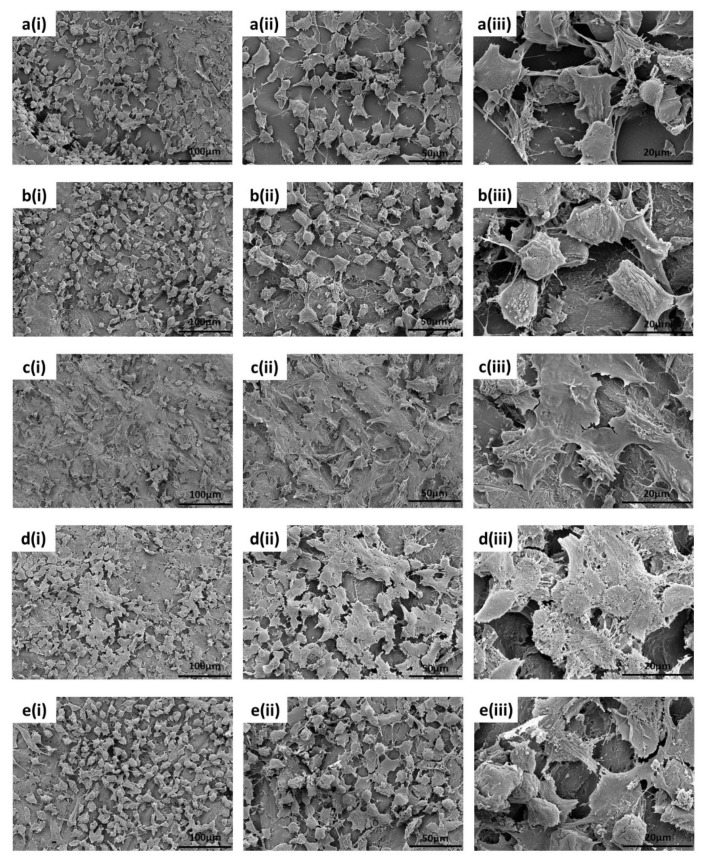
SEM results showing the morphology of U-2 OS cells adhered to the 3D printed samples and cultured under standard conditions for 7 days at magnifications of (**i**) ×300, (**ii**) ×600 and (**iii**) ×2k. Samples (**a**) **i**–**iii** (0HA), (**b**) **i**–**iii** (5HA), (**c**) **i**–**iii** (10HA), (**d**) **i**–**iii** (20HA), and (**e**) **i**–**iii** (30HA).

The morphological evaluation of the adhered osteoblast-like U-2 OS cells on a surface provides a level of complimentary qualitative analysis to the data obtained from the other biological assays such as MTT and PicoGreen™. Figure 14a(i–iii) show the morphological behaviour for the 0HA surface. It is observed that at 7 days, cell spreading is well underway. However, most of the cells viewed at this time point demonstrate a more rounded cell morphology, with filopodia observed to be protruding from all the cells on these 0HA samples. In comparison, the SEM image for the 5HA sample (Figure 14b(i–iii)), shows a similar set of results when compared to the 0HA surface, with the more rounded cell morphology predominating. For the 10HA (Figure 14c(i–iii)), 20HA (Figure 14d(i–iii)), and 30HA (Figure 14e(i–iii)) 3D printed samples the U-2 OS cell exhibit a definite flattened morphology with spread out elongated filipodia. However, for the 30HA sample there did appear to be a high proportion of the U-2 OS cells with a rounded cell morphology when compared to the 10HA and 20HA samples.

## 4. Discussion

The core aim of the work undertaken in this study was to provide a way to regulate concentrations of bioactive HA materials directly on the surface of FFF 3D printed PEEK/HA composite structures in a one step process and to investigate their different chemical, physical, and in vitro properties. Our previous work studied the crystallinity, morphology, bulk properties, and mechanical properties of the same materials and found that the approach taken here could be used to successfully manufacture PEEK/HA composites up to 30 wt% HA [11]. This worked highlighted that the materials were printable, and importantly could be delivered with mechanical properties that matched those of human cortical bone [11]. Other studies have utilised FFF 3D printing of PEEK/HA composites; however, they only investigated the use of up to 10 wt% PEEK/HA and did not achieve the same mechanical properties as those presented here [5,12]. As such, the results presented in this next study deliver the next logical step required in this work, which, to our knowledge, demonstrates the first study of the surface properties of novel FFF 3D printed PEEK/HA composites using advanced surface characterisation techniques such as XPS and ToFSIMS in combination with standard techniques such as XRD, FTIR, and SEM/EDX.

The FTIR results (taken using ATR), shown in Figure 2a highlight that the 3D printed PEEK sample is pure PEEK, with no peaks present from HA and all the peaks with the expected relative intensities for a pure PEEK sample [18]. With the addition of HA into the PEEK matrix, as shown in Figure 2a–d, for the 5HA–30HA samples, respectively, the presence of the PO_4_^3−^ (υ_3_) band (between 1200–900 cm^−1^) is evidence that HA is present in the top 1–2 µm of the surface of the PEEK/HA 3D printed composites (in line with the analysis depth of the ATR techniques utilised here) [32]. As the concentration of HA increases in the composites, the relative intensity of this PO_4_^3−^ band increases significantly with respect to the normalised intensities of the PEEK, indicating a greater prevalence of HA at the surface of these samples as the HA content is increased. This is further corroborated by the XRD results, shown in Figure 3. The pure PEEK sample (0HA) in Figure 3a shows the PEEK to contain no additional phases with all the major reported peaks (highlighted by dashed lines for the (110), (111), (200), and (211) diffraction planes) present at the correct 2θ positions and at the correct relative intensities, as would be expected for pure PEEK [11,18]. After the addition of HA into the PEEK matrix, as shown in Figure 3b–e, for the 5HA–30HA samples, respectively, clear diffractions peaks can be observed for HA (as highlighted in Figure 3), with the peak positions and relative intensities all in line with those expected for HA as outlined in the ICDD file# 09-0432. In addition, the relative peak intensities for the HA material are seen to increase relative to the PEEK as the concentration of HA increases in the samples as shown in Figure 3b–e for the 5HA–30HA samples, respectively. It is also apparent that the diffraction peaks for PEEK or HA show no significant change in their peak width as the concentration of HA increases in the composite materials. The XRD and FTIR results highlight that no new additional phases are present in these 3D printed PEEK/HA composite samples under the conditions employed here [10,25].

The combination here of the high ambient temperature within the print chamber (230 °C), the nozzle temperature (400 °C), and the print-bed temperature (280 °C) are all important to deliver semi-crystalline PEEK/HA composites with high levels of crystallinity as reported previously in our work (44.59–49.91%) [11]. These results are higher than the current reported values in the literature [39]. Previous work by Wu et al. and Zanjanijam et al. showed that as the ambient temperature of the printing chamber is increased, the crystallinity of the polymer matrix increases [40,41]. In such conditions, there are no issues relating to rapid cooling of the printed sample and the polymer chains have adequate time to crystallise, enhancing the overall crystallinity of the samples, as observed here. The nozzle temperature is also very important here for enhancing crystallinity, and that in combination with the higher ambient chamber temperature prevents rapid cooling of the printed specimens, which in turn prevents warping. None of the samples in this study were seen to warp and this is a direct consequence of the high temperatures employed. The print bed temperature is also elevated in this work, well beyond the values reported in previously related experiments by others, although this parameter has received a lot less attention in the literature than the nozzle temperature and the ambient temperature [29,40]. This also helps to prevent warping as it slows down the cooling rate of the polymer when printed. In combination, the temperature of the print nozzle, the print bed, and the print chamber are all critical parameters that collectively ensure the best quality prints without warping and enhanced crystallinity across all the samples. They also act to ensure minimal voids in the printed sample and consistency in the printed layers, as shown in the SEM cross-sections in Supplementary Figure S1. This is a consequence of the elevated temperatures of the print bed and the print chamber, allowing more time for molecular diffusion to occur which results in less surface voids being created. [40,42] However, some small voids are observed in the composite 3D printed samples and suggests that adhesion between the layers is affected by the addition of HA into the PEEK matrix. Consequently, the enhanced crystallinity also delivers enhanced mechanical performance in such 3D printed samples, although that is not the focus of the work in this study. Other parameters that can influence the quality of the print are the nozzle diameter and the feed rate of the filament [11,40]. In this study both the nozzle diameter (1.0 mm) and the feed-rate (40 mm/s) are much higher than previously observed in a range of different studies utilising FFF to 3D print PEEK. Typically, a nozzle diameter of 0.4 mm has been utilised [40,41], although several studies have utilised wider diameter nozzles [40]. For this work, a higher diameter nozzle was employed to prevent clogging of the nozzle with the PEEK/HA composites and to minimise void space in the printed samples. In addition, the printing speed was on the higher end of those seen for FFF 3D printing of PEEK in other studies, although it is comparable to that utilised by Opaldo et al. to 3D print PEEK/HA composites of up to 10% *wt*/*wt* HA in PEEK [5]. However, it should be noted here that each of the different studies utilise different 3D printers, often custom built, each with unique printing capabilities, which sometimes limits the processing conditions that can be employed.

The associated SEM results here also point to increasing levels of HA on the surface of the composite samples, as can be seen from the images in Figure 3. No HA particles can be observed for the pure PEEK (0HA) sample, as shown in Figure 3a(i,ii), but the 5HA through to the 30HA samples all show the presence of HA particles of up to 5µm in diameter. The particles are evenly distributed across the surface of the samples and seem to be more prevalent as the concentration of HA increases in the samples. The FTIR, XRD, and SEM results all show that the PEEK/HA samples have HA homogeneously distributed across the surface of the 3D printed parts and as such present bioactive HA materials that should enhance osseointegration if implanted, in vivo. To confirm that the HA particles are on the uppermost surface of the 3D printed samples, and not just on the sub-surface where they could be detected by the likes of XRD and FTIR, surface sensitive techniques such as XPS and ToFSIMS analyses were also employed in this study. XPS analysis of the different samples clearly showed her that Ca and P species were certainly detectable in the top 10nm of the PEEK/HA composite samples, as highlighted in Figure 5 and Figure 7, with an obvious enhancement of the Ca2p and P2p peak intensities (illustrated in Figure 7c,d, respectively), indicating a greater availability of HA on the uppermost surface of the samples as the HA content in the PEEK/HA composites increases from 0–30 wt%. It is also interesting to note that the Ca/P ratio detected for these samples appears to decrease significantly as the HA content in the composites increases. The reported Ca/P ratios (as highlighted in Table 3) decrease from 2.78 ± 0.87 (5HA) to 1.59 ± 0.09 (30HA). The O/C ratio also increases here (from 0.11 ± 0.00 (5HA) to 0.17 ± 0.00 (30HA)) at the same time. The increase in the O/C ratio is likely to be a consequence of the increasing number of associated PO_4_^3−^ groups, which correlates with the corresponding decreasing Ca/P of the same samples. This can be confirmed as the relative intensity of the lower binding energy O1s peak (around 531.6–531.9 eV) is seen to increase relative to the higher binding energy O1s peak (around 533.6–533.8 eV) in all the PEEK/HA 3D printed samples, as can be observed in Figure 6b and Figure 7b for the 0HA and 30HA samples, respectively. No Ca or P contamination were detected on the surface of the 0HA sample (pure PEEK), and no other impurities were detected using XPS (such as Na), at least within the detection limits of this technique (~0.01 atomic concentration %). It is important to note that ToFSIMS analysis of the same surfaces showed small amounts of contamination across all the 3D printed PEEK and PEEK/HA composites, with the likes of Na, F and K observed, along with a range of organic species that would not be associated with PEEK, namely C_2_H_3_^+^, C_2_H_4_^+^, and (C_2_H_5_^+^) as typical examples. These can clearly be observed across all the positive ion ToFSIMS spectra in Figure 8a 0HA and Figure 8b 30HA, Specific peaks with a high intensity included *m/z* 39 and 51, are indicative of either aromaticity or ionically diagnostic of PEEK/PEEK fragments by Pawson et al. [37] and are clearly shown across all the 3D printed samples. Ca^+^, CaH^+^ and CaOH^+^ peaks can be observed at *m/z* of 40, 41, and 57 on all samples. However, when the normalised peak intensities for all the samples are calculated, as shown in Figure 9, the concentrations of these Ca species are negligible in the 0HA (pure PEEK sample) when compared to the PEEK/HA 3D printed composites. Higher concentrations of Ca^+^, CaH^+^ species are observed in all the PEEK/HA composites when compared to CaOH+, with the 20HA sample reporting the highest concentrations of Ca^+^ and CaH^+^ species. It would have been expected here that these concentrations would have been higher in the 30HA sample; however, small variations in the concentration of the HA in the extruded filament or slight variations in the 3D printing environment could play a role in this anomaly and need to be further investigated. Despite this, the distribution of Ca species (Ca^+^, CaH^+^ and CaOH^+^) are all observed to be homogenous across the entire area of the samples analysed. This indicates that the Ca species in HA is available in the uppermost surface regions of these samples (1–2 nm), which is the desired outcome. Previous studies on PEEK/HA composites manufactured using extrusion free forming [1] and compounding and injection moulding [13] have shown that such materials are more favourable for implantation than pure PEEK materials, due to the addition of bioactive HA.

In vitro testing of the different 3D printed composites revealed that all the samples support the attachment, growth, and viability of U-2 OS osteoblast-like cells for at least 7 days. The 30HA 3D printed sample had the highest cellular metabolism at 7 days when compared to the pure PEEK samples (0HA), which may be a consequence of the higher HA content in this sample, as highlighted in Figure 12. However, it should be noted that MTT records cellular metabolism and not cellular viability or proliferation directly, therefore, it cannot be ruled out that the cellular metabolism observed for the 5HA–20HA samples here is a consequence of touch contact inhibition [43]. The cell shape (morphology) is highly indicative of the osteoblast-like cell behaviour in relation to both adhesion and viability, as it should be observed that a cell that has a positive interaction with suitable surface properties will show signs of cell spreading on the surface. The corresponding SEM images of the U-2 OS cells at 7 days, as shown in Figure 14, highlights that cell are adhering and spreading on all the samples; however, the 0HA, 5HA, and 30HA samples have more visibly rounded cells than the 10HA and 20HA samples. This suggests that the cells have not fully interacted with their surroundings, and full cell attachment has not occurred yet at this time-point. Filopodia are seen to be protruding from the cells on all the 3D printed samples (as highlighted in Figure 14), indicating that the cells have begun to probe the underlying substrate for topographical features to further guide their attachment. For the 10HA and 20HA samples, the U-2 OS cell exhibit a definite flattened morphology when compared to the 0HA, 5HA, and 30HA samples and appeared to adhere securely to the substrate surface with spread out elongated filipodia. However, for the 30HA sample there did appear to be a high proportion of the U-2 OS cells with a rounded cell morphology when compared to the 10HA and 20HA samples. The 30HA sample does show a significant proportion of flattened cells as well. Cell viability and proliferation testing using the PicoGreen™ assay, shown in Figure 13 highlighted that cells adhered well to all the 3D printed samples at 7 days and corroborates the results observed in the MTT and cell morphology tests at the same timepoint. In particular, the 5HA and 10HA samples showing higher DNA concentrations than pure peek (0HA) and are therefore exhibiting enhanced U-2 OS viability and proliferative capacity. It has been suggested that with an increasing HA content in the 20 HA and 30HA samples that there may elevated levels of free Ca^2+^ in the surrounding cell culture medium due to dissolution of the HA, therefore this could inhibit cellular proliferation [10]. This may explain the slightly lower DNA concentrations detected here via the PicoGreen™ assay. The lower proliferative capacity may also indicate the onset of osteoblast differentiation in the 20HA and 30HA at this early timepoint. There appears to be no correlation here between the surface roughness, surface morphology or contact angle with respect to the MTT, PicoGreen™ assays, or cell morphology results. This could have been a consequence of the temperatures of the colling composite material, with small differences perhaps introducing subtle changes to the surface chemistry and morphology and influencing the results. It is obvious that the processing conditions here, namely the control of the temperature may need studies more accurately and possibly in real-time to provide a means of altering the printing conditions in situ and removing the possibility of inconsistency in the surface properties of the samples produced. It has been reported that the surface roughness does decrease with increasing nozzle temperatures in 3D printing [42]. The contact angle for the 0HA is low at 43.56 ± 4.11°, lower than might be expected for pure PEEK as determined in other studies [4,10,44]. Further to this the water contact angle is seen to increase significantly for all the 3D printed PEEK/HA composites, with values in the range 77.40 ± 10.03° − 93.08 ± 5.44°, highlighting a tendency towards less hydrophilic surfaces, with the 30HA being borderline hydrophobic [30]. It was shown that the contact angle can reduce significantly when agents are added to the PEEK matrix to manufacture composites [10,13,44,45]. However, there are a range of conflicting results reported in the literature with respect to the water contact angle of PEEK/HA bio-composites. Some studies have highlighted a slight increase in the contact angle after the addition of HA to PEEK/HA biocomposites, whilst others report a decrease in the water contact angle with the addition of HA particles to PEEK [10,44,45,46,47]. Whereas it would be expected that the water contact would increase with the addition of HA particles on the surface of the sample, localised surface properties and the surface topography can heavily influence this [46]. Typically, the adhesion and proliferation of osteoblasts has been associated with good wettability (hydrophilicity), with the osteoblast cells exhibiting strong preference for hydrophilic surfaces [44]. All the samples here were hydrophilic in nature, with only the 30HA sample showing borderline hydrophobicity. Notwithstanding this in the results reported here, the sample surfaces are quite rough, and this may have had an influence on the measured contact angles and merits more detailed examination. Porosity is also known to influence the osteoblast response, more so than any associated surface roughness, as highlighted by Spece et al. [48]. Despite these findings, it could be suggested that the HA content on the surface of the samples has the most significant influence on the in vitro behaviour observed in this study, which has been previously suggested for PEEK/HA biocomposites [1,2,3,5,13]. For all samples, the results do indicate healthy cell functioning and a positive cell-surface response between the U-2 OS cells and the 3D printed samples up to 7 days, and further in vitro testing is required to discriminate between the potential bioactivity of these different FFF 3D printed PEEK/HA composites. These will include the consideration of the initial cellular adhesion events and in-depth consideration of cellular differentiation to determine their suitability going forward for orthopaedics. These results highlight that 3D printing is a useful tool for printing PEEK and HA composites, and that the printing technology is developing to enable the manufacture of customised medical devices in the future [49,50,51].

## 5. Conclusions

In this work we reported the direct fused filament fabrication 3D printing of PEEK/HA composites with up to 30 wt% HA content and their subsequent surface characterisation. Surface characterization of such materials is a critical consideration, as it is important to understand the relationship between the surface properties and the subsequent biological response, as it is the surface that will ultimately guide their osteointegration. The key aim of the work was to provide a one-step additive manufacturing route to deliver PEEK/HA composites with tunable concentrations of HA on the surface of the 3D printed structures without the need for any further processing steps to expose the bioactive HA materials. A custom modified commercial Ultimaker 2+ (UM2+) 3D printer was employed, which was updated to operate with the print chamber temperature up to 230 °C, the print bed up to 350 °C, and the hot-end printing nozzle up to 420 °C. The results here clearly demonstrate through surface chemical and physical analyses that on the surface region of the PEEK/HA composite samples had a high degree of crystallinity, as highlighted by the XRD results. In addition, the SEM/EDX, FTIR, XPS, and ToFSIMS all confirm the presence of HA in the uppermost surface regions of the samples without the need to modify the surface. As the content of the HA increased in the filaments (from 0 wt% HA to 30 wt% HA) more HA is observed on the surface of the samples, with a decreasing Ca/P ratio observed as the HA content increases. From the in vitro characterisation all the sample surfaces 3D printed here support the adherence and growth of viable U-2 OS osteoblast like cells up to 7 days. Any subtle differences in the cellular response seems to be related to the HA content on the surface of the samples as opposed to any physical characteristics of the different materials. As such, these results highlight that the 3D printed PEEK/HA composites manufactured here can have the concentrations of HA controlled on their uppermost surface in a one-step process, and that this approach provides a route for delivering bioactive agents that have the potential to enhance direct bone apposition in orthopaedic implants, such as spinal fusion devices.

## Figures and Tables

**Table 1:** The 3D Printing parameters.

Description	Value
Nozzle diameter	1.0 mm
Layer Thickness	0.1 mm
Nozzle temperature	400 °C
Building plate temperature	280 °C
Chamber temperature	230 °C
Printing speed	40 mm/s
Raster angle	XY 45°/−45°

**Table 2:** XPS peak positions from peak fitting.

Peak	Sample				
	0HA	5HA	10HA	20HA	30HA
**O1s**	531.6	531.7	531.9	531.9	531.5
**O1s**	533.8	533.7	533.8	533.6	533.6
**Ca2p_3/2_**	-	347.6	347.6	347.6	347.6
**Ca2p_1/2_**	-	350.9	351.1	351.1	351.1
**C1s**	285.0	285.0	285.0	285.0	285.0
**C1s**	286.6	286.7	286.5	286.6	286.4
**C1s**	287.4	287.3	287.2	287.1	287.3
**C1s**	289.2	-	-	288.8	288.9
**C1s**	291.8	291.8	291.9	291.7	291.7
**P2p**	-	133.6	133.6	133.7	133.7

**Table 3:** Atomic Concentration %, Ca/P and O/C as determined by XPS.

Sample	C	O	Ca	P	Ca/P	O/C
0HA	87.85 ± 0.76	12.15 ± 0.76	-	-	-	0.14 ± 0.01
5HA	89.70 ± 0.10	9.92 ± 0.08	0.27 ± 0.03	0.11 ± 0.04	2.78 ± 0.87	0.11 ± 0.00
10HA	89.50 ± 0.30	9.76 ± 0.27	0.49 ± 0.06	0.24 ± 0.05	2.22 ± 0.81	0.11 ± 0.00
20HA	86.62 ± 0.50	12.29 ± 0.51	0.69 ± 0.07	0.40 ± 0.10	1.91 ± 0.69	0.14 ± 0.01
30HA	83.49 ± 0.27	14.44 ± 0.25	1.29 ± 0.03	0.81 ± 0.03	1.59 ± 0.09	0.17 ± 0.00

## Data Availability

The raw/processed data required to reproduce these findings are available from the authors on request.

## Supplementary Materials

The following are available online at https://www.mdpi.com/article/10.3390/polym13183117/s1, Figure S1: Cross-sectional BSE SEM images of 3D printed (a) 0HA, (b) 5HA, (c) 10HA, (d) 20HA and (e) 30HA. Scale bar = 500 µm, Figure S2: Surface Roughness of the 3D printed samples (R_a_). (µm). N=4 for each sample type, Figure S3: XPS high resolution scans for 5HA where a (C1s), b (O1s), c (Ca2p), and d (P2p), Figure S4: ToFSIMS spectrum for 5HA, Table S1: Surface Roughness of the 3D printed samples (R_a_). (µm). n = 4 for each sample type, Table S2: Water Contact Angle of the 3D printed samples (°). n = 6.
