# Supplementary material for: The Surface Characterisation of Fused Filament Fabricated (FFF) 3D Printed PEEK/Hydroxyapatite Composites
Source: Polymers (Basel). 2021 Sep 15;13(18):3117. doi: 10.3390/polym13183117 (PMC8471434; doi:10.3390/polym13183117)
Supplement: Supplementary file 1 [file polymers-13-03117-s001.zip › polymers-1331072-supplementary.pdf]

Article

The Surface Characterisation of Fused Filament Fabricated (FFF) 3D Printed PEEK/Hydroxyapatite Composites

Krzysztof Rodzeń [1,*], Mary Josephine McIvor [1], Preetam K. Sharma [1,2], Jonathan G. Acheson [1], Alistair McIlhagger [1], Mozaffar Mokhtari [1], Aoife McFerran [1], Joanna Ward [1], Brian J. Meenan [1] and Adrian R. Boyd [1,*]

[1] School of Engineering, Ulster University, Shore Road, Newtownabbey BT37 0QB, UK;
mj.mcivor@ulster.ac.uk (M.J.M.); p.sharma@lboro.ac.uk (P.K.S.);
j.acheson@ulster.ac.uk (J.G.A.); a.mcilhagger@ulster.ac.uk (A.M.);
m.mokhtari@ulster.ac.uk (M.M.); mcferran-a4@ulster.ac.uk (A.M.);
je.ward@ulster.ac.uk (J.W.); bj.meenan@ulster.ac.uk (B.J.M.)

[2] Department of Chemical Engineering, Loughborough University, Loughborough LE11 3TU, UK

* Correspondence: kp.rodzen@ulster.ac.uk (K.R.); ar.boyd@ulster.ac.uk (A.R.B.); Tel.: +44-(0)-289-036-8924 (A.R.B.)

Supplementary Information

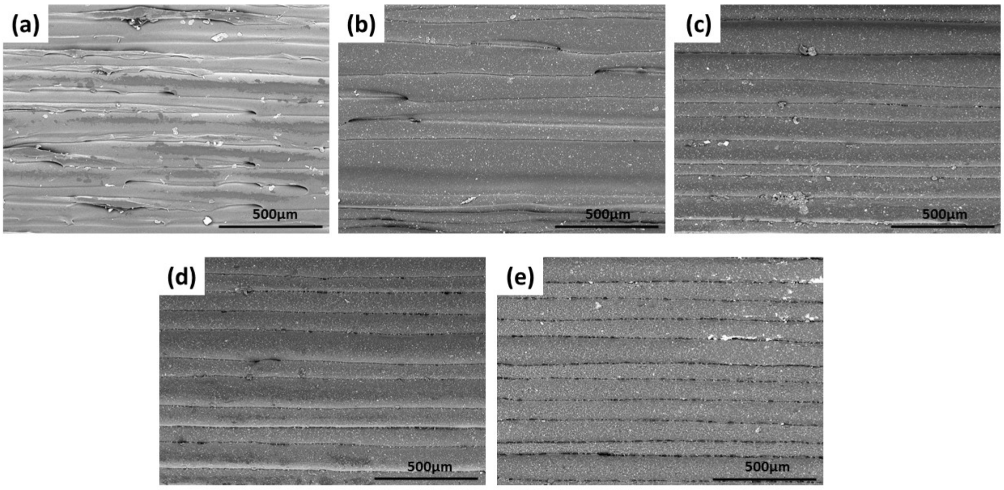

Figure S1. Cross-sectional BSE SEM images of 3D printed **(a)** 0HA, **(b)** 5HA, **(c)** 10HA, **(d)** 20HA and **(e)** 30HA. Scale bar = 500 μm.

Table S1. Surface Roughness of the 3D printed samples (R_a). (μm). n = 4 for each sample type.

Sample	(R_a) (μm)
**0HA**	3.15 ± 0.44
**5HA**	4.41 ± 0.53
**10HA**	2.70 ± 0.57
**20HA**	3.37 ± 0.47
**30HA**	3.40 ± 0.65

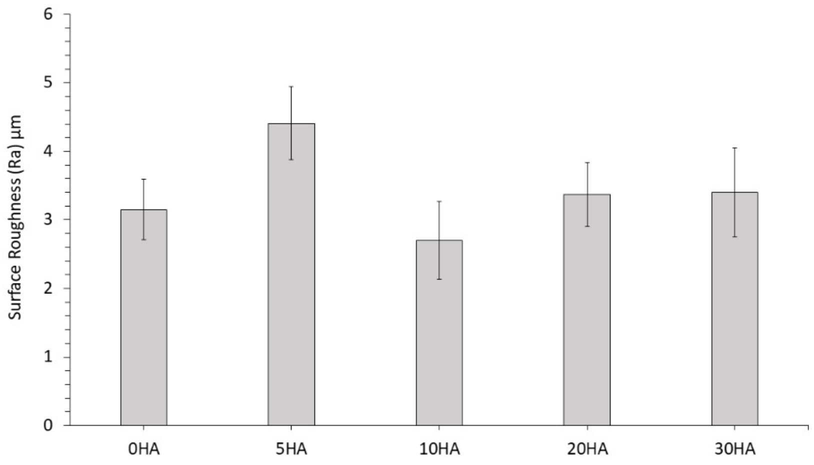

Figure S**2.** Surface Roughness of the 3D printed samples (R_a). (µm). N=4 for each sample type.

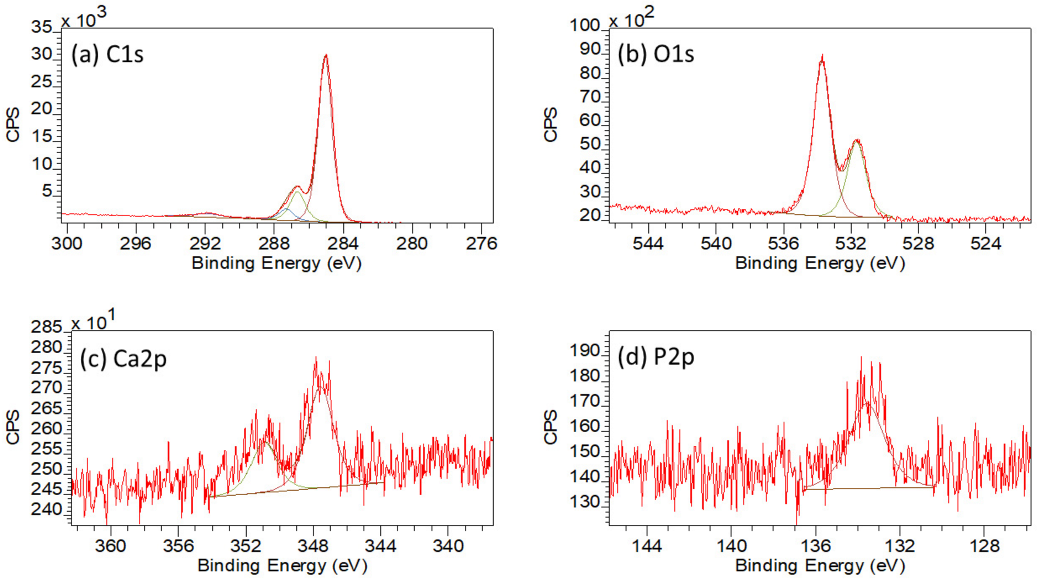

Figure S**3.** XPS high resolution scans for 5HA where a (C1s), b (O1s), c (Ca2p), and d (P2p).

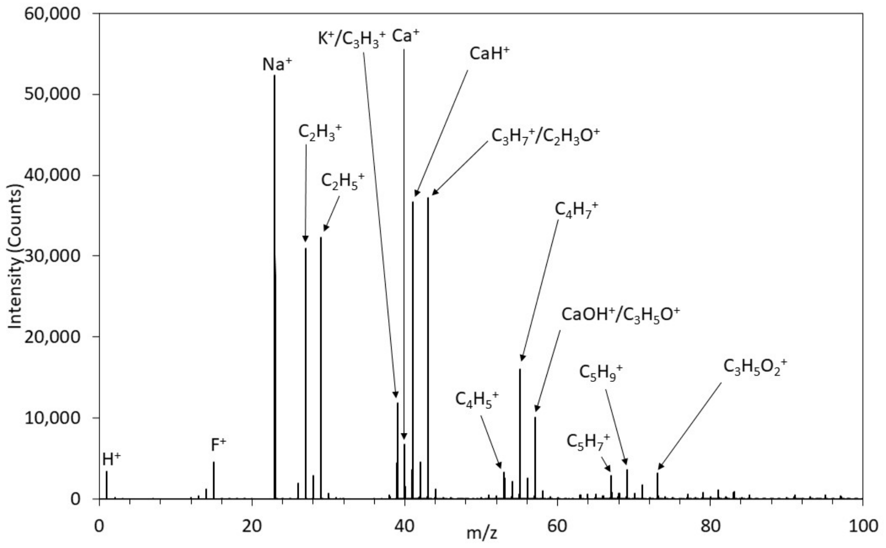

Figure S**4.** ToFSIMS spectrum for 5HA.

Table S**2.** Water Contact Angle of the 3D printed samples (°). n = 6.

Sample	Water Contact Angle (°)
0HA	43.56 ± 4.11
5HA	82.19 ± 5.95
10HA	77.40 ± 10.03
20HA	90.08 ± 4.32
30HA	93.08 ± 5.44
